# Phyllodes Tumors of the Breast: A Review of 26 Cases

**DOI:** 10.4021/wjon2010.06.220w

**Published:** 2010-05-19

**Authors:** Soumaya Ben Abdelkrim, Amel Trabelsi, Mouna Bouzrara, Mohamed Zaher Boudagga, Anis Memmi, Dajla Abbassi Bakir, Moncef Mokni

**Affiliations:** a Department of Pathology, Farhat Hached Hospital, 4000, Sousse, Tunisia; b Department of Medical Oncology, Farhat Hached Hospital, 4000, Sousse, Tunisia; c Department of Gynecology, Farhat Hached Hospital, 4000, Sousse, Tunisia; d Department of Radiology, Farhat Hached Hospital, 4000, Sousse, Tunisia

**Keywords:** Phyllodes tumor, Pathology, Prognosis, Treatment, Outcome

## Abstract

**Background:**

Phyllodes tumors of the breast are rare and locally aggressive neoplasms. Our study aimed to report the experience of the Farhat Hached Hospital (Sousse, Tunisia) acquired during a 7-year period and to give an additional review of the available literature.

**Methods:**

The authors analyzed retrospectively clinical, radiological, histopathological and therapeutic features as well as outcome in a series of 26 cases diagnosed as phyllodes tumors of the breast at the Pathology Department of Farhat Hached Hospital, Sousse, Tunisia, from 2003 to 2009. The slides were reviewed in order to classify the tumors into benign, borderline and malignant on the basis of the criteria proposed by the World Health Organization.

**Results:**

All the cases occurred in women. The analysis of this series showed the following characteristics: mean age at diagnosis was 40 years (19 - 66), tumor size was 1.5 - 40 cm (mean: 7.8 cm); the chief complaint was a mammary mass; the right breast was affected in 14 cases, the upper outer quadrant was the most commonly involved site (42.3%); surgical treatment was used in all cases, 21 patients (80.8%) were treated conservatively (13 benign, 6 borderline, and 2 malignant) and 5 (19.2%) by radical surgery (1 borderline and 4 malignant); seven patients underwent post-operative radiotherapy; in 14/19 cases (73.7%), a good correlation was observed between intraoperative frozen section analysis and definitive histology; the tumor was classed as benign in 13 cases (50%), borderline in 7 cases (27%) and malignant in 6 cases (23%); follow-up data was available in 22 cases; the rate of recurrence was 23% (1 benign, 3 borderline, and 2 malignant) after a mean follow-up of 13.6 months; all the recurrent tumors were initially treated by lumpectomy and were close to margin at the initial pathologic examination; the treatment of recurrences consisted of simple mastectomy in 5 cases, and local excision in one case; three patients developed metastases, one of whom after recurrence**;** three patients have died.

**Conclusions:**

This is a substantial single institution experience of a rare tumor. Phyllodes tumors of the breast have an unpredictable outcome, thus a wide local excision, with an adequate margin of normal breast tissue, is the preferred initial therapy.

## Introduction

Phyllodes tumors (PTs) of the breast are uncommon tumors showing a variable clinicopathological behavior ranging from an apparently benign course to malignancy [1]. Clinicopathological, therapeutic and outcome data of a series of 26 patients diagnosed with a phyllodes tumor (PT) of the breast are reported. A short review of diagnosis, histology, treatment and prognosis of PT follows.

## Patients and Methods

We retrospectively reviewed the medical records of a series of 26 patients diagnosed with a PT of the breast at Farhat Hached Hospital (Sousse, Tunisia) during a 7-year period from 2003 to 2009. The extracted data included age at the time of diagnosis, the duration of the illness, personal and family history, presenting symptoms, tumor size, tumor localization, mammography findings, echography findings, preoperative diagnosis, surgical procedure, pathological findings and outcome. Grading into benign, borderline and malignant was performed on hematoxylin and eosin (HE) stained sections, using the 2003 World Health Organization (WHO) classification taking into account stromal cellularity and overgrowth, cellular atypia mitotic activity, and microscopic tumor borders (circumscribed or infiltrating) [1].

## Results

The patients were ranging in age from 19 to 66 years (mean age: 40 years). The mean age of occurrence of benign, borderline and malignant tumors was 35.8, 44.7 and 45.2 respectively. Breastfeeding was noted in 14 out of 17 patients who had children. Only 5 women were menopaused at the time of diagnosis. Six patients had antecedents of fibroadenoma of the same breast and 2 patients had a history of fibrocystic disease with epithelial hyperplasia of the same breast, while 3 patients had a family history of breast cancer. Only one patient was pregnant at diagnosis. Four patients had used Contraceptive agents. Patients presented with a palpable breast mass in 25 cases, pain was present in 8 cases, and cutaneous signs (skin fixation, nipple retraction, ulceration of the overlying skin) were seen in 9 cases.

**Figure 1:**
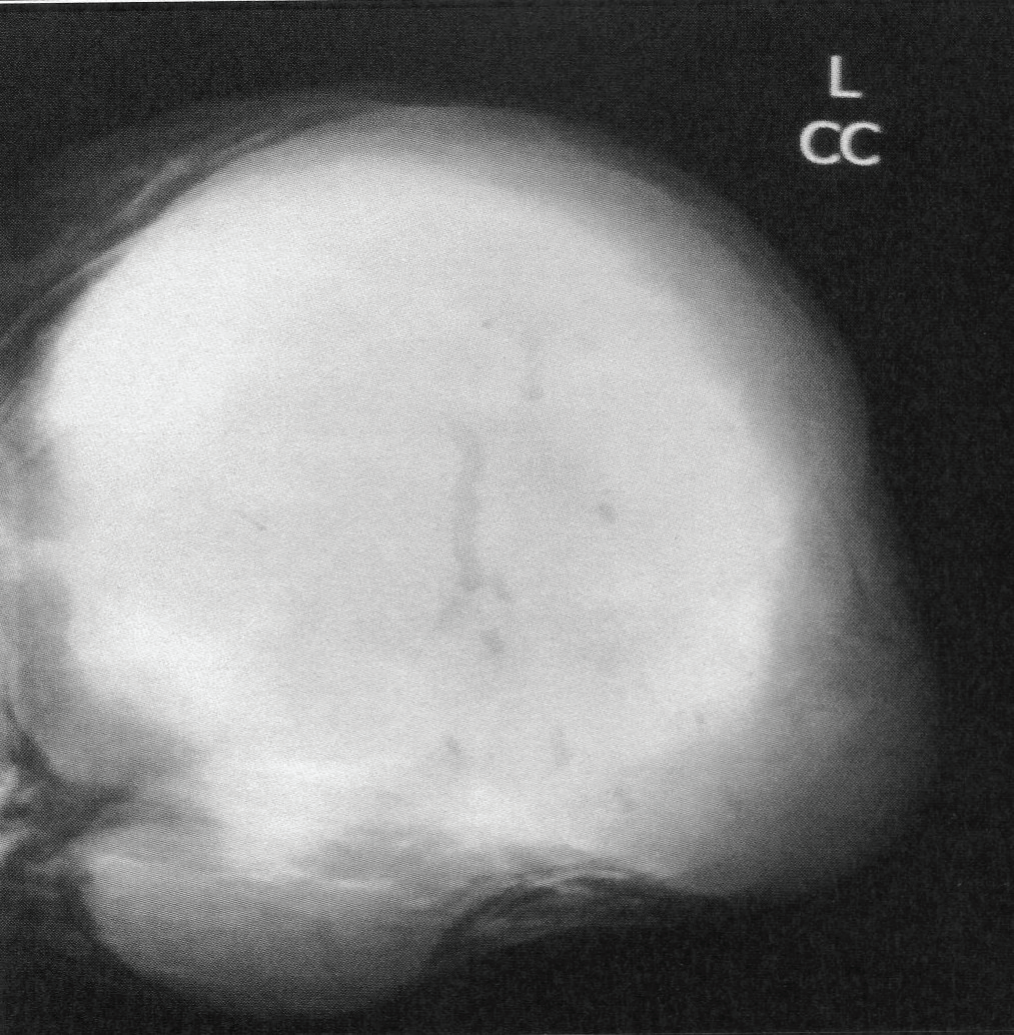
Mammography revealing a lobulated mass with smooth margins.

**Figure 2:**
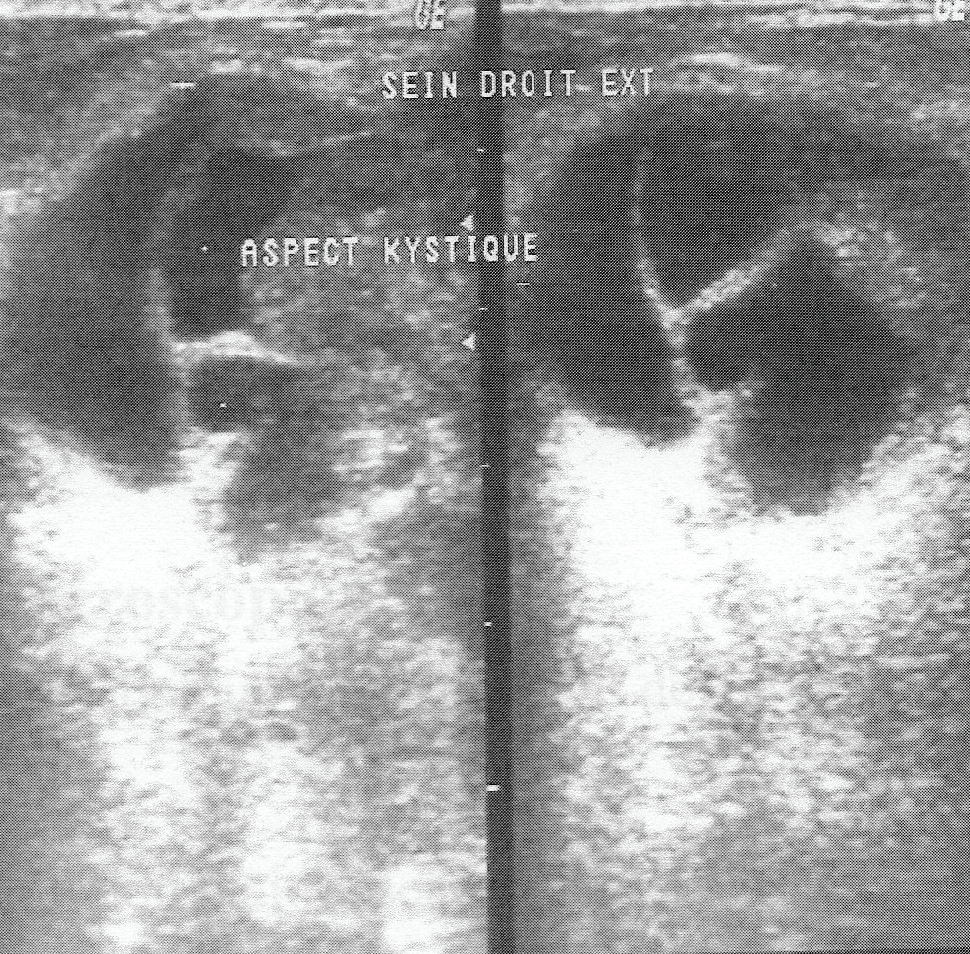
Ultrasound of the breast revealing a solid smooth bordered mass with cystic spaces.

The mean time from onset of symptomatology and pathological diagnosis of PT ranged from 1 to 96 months (mean 20 months). The tumors occurred in the left breast in 12 cases, in the right one in 13 cases and in the remaining case, it was bilateral. The upper outer quadrant was involved in 11 cases (42.3%). Mammography was made in 25 cases and showed a well defined opacity (Fig. 1) in 14 cases, suggesting the diagnosis of PT in 8 cases. In 11 cases, mammography showed a poorly circumscribed mass suggestive of malignancy. Echography, performed in all the cases, showed a heterogeneous, hypoechoic, well delineated, and cystic mass (Fig. 2) suggesting a PT in 16 cases. In 4 cases, the diagnosis of fibroadenoma was suggested and in 6 cases, it was doubtful between PT and fibroadenoma. Fine needle aspiration was performed in 4 cases and suggested the diagnosis of PT in only one case.

**Figure 3:**
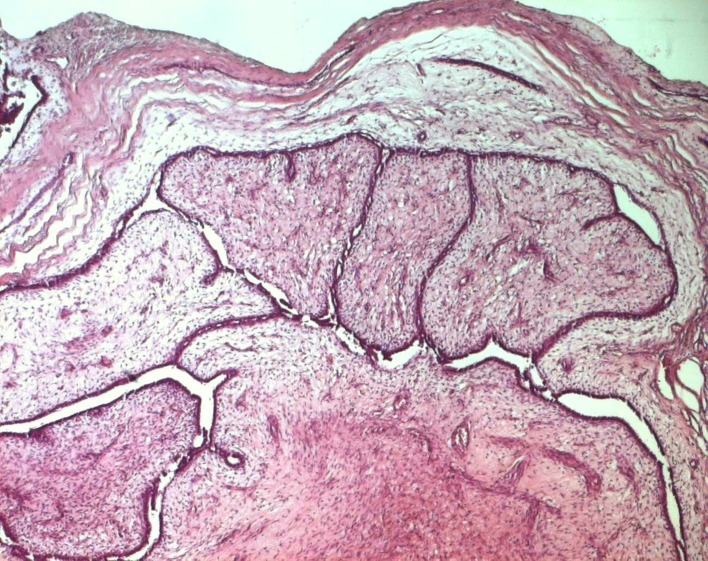
Benign phyllodes tumor: a well-circumscribed biphasic neoplasm containing leaf-like, epithelial-lined papillary projections penetrating into cystic spaces (HE x 40).

**Figure 4:**
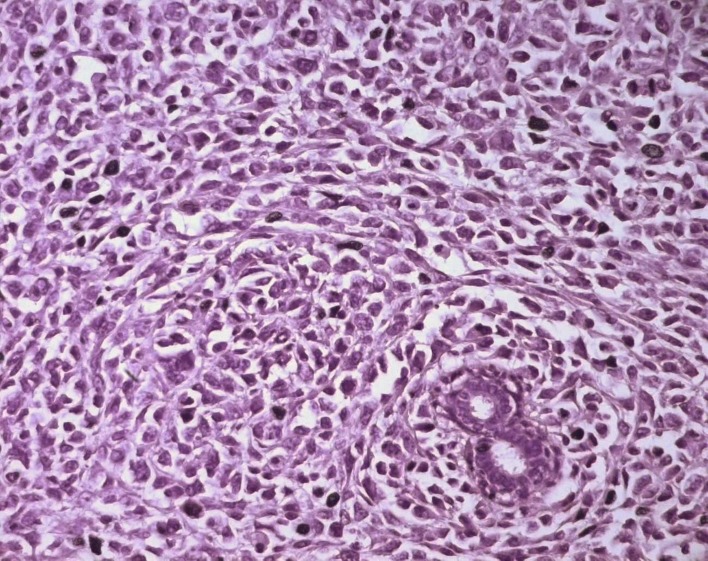
Borderline PT consisting predominantly of a stromal overgrowth, with some ducts (HE x 400).

**Figure 5:**
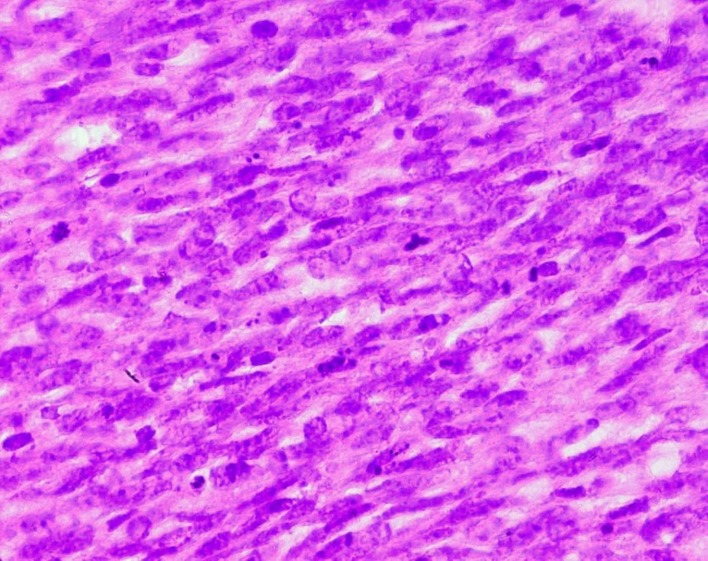
Stromal cells of a malignant PT showing marked nuclear atypia and frequent mitoses (HE x 400).

Surgical treatment was primarily applied in all cases, consisting in lumpectomy in 21 cases and total mastectomy in 5 cases (4 malignant PTs and 1 borderline PT). Frozen-section analysis was undertaken in 19 cases. It was concordant with final histology in 14 cases. Three out of the 5 remaining cases were benign PTs at the final histology and assessed as fibroadenomas in frozen-section analysis. One borderline PT at the final histology was assessed as malignant PT at frozen-section analysis and one borderline PT was incorrectly assessed as benign at frozen-section analysis. On gross appearance, tumors ranged in size from 1.5 to 40 cm (mean 7.8 cm), 14 tumors were larger than 5 cm. The size range for benign, borderline, and malignant tumors was 1.5 to 40 cm (mean: 7.4 cm), 1.7 to 15 cm (mean: 7.5 cm), and 3.6 to 23.4 cm (mean: 9 cm), respectively. On histological examination, 13 tumors were graded as benign (Fig. 3), 7 tumors as borderline (Fig. 4) and 6 tumors as malignant (Fig. 5).

Table 1 summarizes the population characteristics by PT grade. The growth pattern was expansile with relatively fair circumscription in 19 cases, and in the 7 remaining cases, tumors were poorly circumscribed with infiltrative margins. Satellite micronodules were seen in 3 cases (2 malignant and 1 borderline). Cystic changes were seen in 4 cases. Foci of necrosis and hemorrhage were observed in 4 cases. The resection margin was recorded as negative (tumor was more than 1 cm from the margin) in 14 cases, and close (the tumor was within 1 cm of the margin) in 10 cases, while adequate assessment of resection margins could not be performed in 2 cases because of fragmented specimens. Seven patients underwent post-operative radiation (2 borderline and 5 malignant PTs). Four patients were lost to follow-up after surgery, and the follow-up period of the remaining 22 patients ranged from 5 to 33 months with a mean of 13.6 months. Six patients experienced local recurrence at the same site in an average period of 7.6 months. Local recurrence was found in 2 malignant cases, 3 borderline PTs and one benign PT.

**Table 1:** Population Characteristics by PT Grade

	Benign	Borderline	Malignant
Number of patients	13	7	6
Mean age (range)	35.8 (19 - 55)	44.7 (34 - 52)	45.2 (29 - 66)
Size			
< 5 cm	6	2	3
≥ 5 cm	7	5	3
Surgical approach			
breast-conserving surgery	12	6	2
mastectomy	1	1	4
Surgical margin			
negative	6	4	4
close	5	3	2
indeterminate	2	0	0
Outcome			
Recurrence	1	3*	2
Metastases	0	1*	2
Favorable	10	4	0
Lost to follow-up	2	0	2

*One patient developed recurrent disease at 10 months and lung metastases at 21 months.

All these tumors were initially treated by lumpectomy and were close to margin at the initial pathologic examination, and they were all of the same histological grade as the one of the primitive tumor. Treatment included lumpectomy in the case of recurrent benign PT, and mastectomy in the 5 other recurrent cases, combined with post-operative chemotherapy in 2 cases, with both chemo- and radiotherapy in 2 cases, and with only post-operative radiotherapy in one case. Lung metastases occurred in 3 patients, one of whom had already local recurrence, metastatic tumors developed in an average period of 24 months and were treated by radio- and chemotherapy. Three patients died during the follow-up period because of advanced metastatic disease in 2 cases and from chemotherapy complications in one case.

## Discussion

Phyllodes tumor is a rare fibroepithelial breast neoplasm with unpredictable clinical course, which resembles fibroadenoma; it accounts for 0.3% to 1% of all primary breast tumors and 2.5% of fibroepithelial breast lesions [1]. Fibroadenomas account for almost all of the remaining fibroepithelial tumors [2]. PTs have been first described by Johannes Muller in 1838 [3] as cystosarcoma phyllodes, based on the tumor’s ‘leaf-like’ projections into cystic spaces and sarcomatous stroma. This term is misleading and has since been discouraged, as more than 70% of these lesions follow a benign course and only rarely exhibit cystic degeneration [4]. PTs were considered benign until the first reported case of a metastatic tumor in 1931 [5]. This tumor has in fact a very variable but usually benign course, and it has a propensity to locally recur and the ability to metastasize.

The median age group in which these tumors occur (45 years) is about 15 years older than the age group for fibroadenomas [6]. PTs often present as palpable masses, most commonly located in the upper outer quadrant of the breast. In the series of Barrio et al, including 293 cases, 3.4% of the patients had bilateral PT [7]. In our series, only one bilateral PT was seen (3.8%). PTs usually grow slowly and are often painless. Nipple retraction and bloody nipple discharge may occur when the tumor involves the areolar region [8, 9]. PTs vary greatly in size with a mean size of 4 - 5 cm, larger tumors are more likely to be malignant, but there are many exceptions [2, 6].

Mammography and ultrasound appearances are non-specific and the pre-operative diagnosis of PT is difficult since rapid growth and/or large size of apparent fibroadenomas may be the only imaging findings suggesting PT [10]. PTs appear on mammography as lobulated round or oval masses with well-circumscribed borders and rarely contain calcifications [11-13]. On sonography, PTs are usually well-defined, solid masses with heterogeneous internal echoes, without posterior acoustic attenuation. A diagnosis of PT should be considered if sonography reveals fluid-filled, elongated spaces or clefts in a solid mass. It is often difficult to differentiate PT from fibroadenoma on sonography or mammography, and it is not possible to distinguish between benign and malignant PTs on the basis of sonographic or mammographic findings [9]. Magnetic Resonance Imaging may be used to delineate the full tumor extent and potential satellite lesions before surgical excision [13]. PTs can occur synchronously with fibroadenoma with an incidence higher than the percentage seen in the general population [7]. The percentage of concurrent fibroadenomas varies from 4.2% [14] to nearly one third of women with PTs [10].

Histologically, PTs of the breast are biphasic fibroepithelial tumors composed of epithelial elements arranged in cleft-like ducts surrounded by a predominant connective tissue stroma typically organized in leaf-like structures. This mesenchymal component shows morphologic patterns that range from fibroadenoma-like to frankly sarcomatous [1, 4]. Regressive changes may appear, such as necrosis, cysts and bleeding [15]. Currently, PTs are classified into benign, borderline and malignant based on microscopic features consisting in stromal cellularity, cellular pleomorphism, mitotic activity, margin appearance (circumscribed vs. permeative margins), and stromal overgrowth (pronounced proliferation of stromal components relative to glandular structures, defined as at least one 40 power field of stroma without epithelium) [1, 16]. In 19 series of PTs, benign, borderline and malignant PTs ranged between 35% - 85%, 7% - 40% and 7% - 45% respectively [2]. In our series, these percentages were respectively 50%, 27% and 23%. Malignancy develops in the mesenchymal component of PT, whereas the ductal component is usually benign, and though ductal elements may occur in local recurrences, metastatic lesions consist of only the sarcomatous component [17]. PTs that harbor carcinoma are usually benign, and it has been stated that when a malignant PT contains carcinoma, it becomes, by definition, a carcinosarcoma or metaplastic carcinoma, as the tumor is composed of carcinomatous and sarcomatous elements [6]. Many histological prognostic factors have been evaluated to identify tumors with potentially aggressive behavior. Aggressive pathologic features include grade [8, 14, 18, 19], stromal overgrowth [20, 21], infiltrative borders, marked stromal overgrowth [22], stromal atypia [14, 23], marked stromal cellularity [14], high mitotic count [22, 23], pseudoangiomatous stromal hyperplasia [14], heterologous stromal elements, fibroproliferation in the surrounding breast tissue [7] and tumor necrosis [7, 23, 24]. Some authors, however, questioned the usefulness of prognostic assessments based solely on histologic classification [14, 25]. In fact, histologically benign PTs have been reported to have metastasized [8, 26] and many histologically malignant PTs neither recur nor metastasize [4, 25]. The imperfect correlation of histological findings with the subsequent clinical course highlights the need for molecular markers that can more reliably predict patient's outcome. Recently described methods include immunohistologic assessment of p53, Ki-67, CD34, factor XIIIa, c-myc and c-Kit but their prognostic usefulness has not been affirmed yet [4, 14]. The benign or malignant nature of a locally recurrent PT is generally identical to the primary neoplasm, as in our series. However, there are several instances in the literature that document the ability of benign tumors to undergo malignant transformation [7]. Reinfuss et al [8] state that this transformation is related to the presence of malignant foci in the primary specimen that were missed on pathologic examination. The histological downgrading of recurrent tumors is unusual and was reported in 6 cases in the study of Tan et al [14]. A likely explanation is insufficient sampling of recurrent lesions in this series. Pathological diagnosis of PTs can be difficult on cytology or small biopsy specimens: PTs are often heterogeneous such that some areas of the tumor are indistinguishable from fibroadenoma and a small biopsy may only sample these areas. Furthermore, biopsies of a malignant PT may include only the malignant stromal component of the tumor and a diagnosis of sarcoma may be rendered if the biphasic nature of the tumor is not appreciated [2]. Thus, confidence in correctly diagnosing the lesion is significantly greater with excisional biopsy [11].

Standard therapy includes wide surgical excision with a margin of more than 1 cm even when pathologic features suggest benignity. Mastectomy is necessary only when tumor cannot be removed with adequate clearance [2, 4, 6]. Most of the studies in the literature have found that a positive margin status is the most consistent indicator of local recurrence [2, 7, 14, 27]. Preoperative diagnosis is then important for good local control. Wide reexcision should be considered when the margins are involved microscopically [27]. In a recent large series [24], total mastectomy for the malignant and borderline tumors had better results than breast conserving surgery. In that study, RT delivery was an independent favorable prognostic factor for local control of the malignant and borderline group. Metastasis in PT usually spread hematogenously to the lungs, pleura, or bone [10, 11] and axillary lymph node dissection is not indicated [6, 28]. Adjuvant systemic therapy is of no proven value [29]. Patients with locally recurrent disease should undergo wide excision of the recurrence [29]. The recurrence rate reported by 19 studies of PTs varied widely (benign 1.5% - 12%, borderline 0% -38.5% and malignant 3% - 50%) but has an upward trend with increasing grade [2]. The WHO reported an overall metastatic rate of 10% (benign 0%, borderline 4% and malignant 22%) [1]. The range of 5-year disease survival is 78% - 91% [2].
